# Radiation exposure in trauma patients is affected by age

**DOI:** 10.1186/cc13229

**Published:** 2014-03-17

**Authors:** M Wagner, JV Vonk, CW Wichman, AH Hegde, JO Oliveto

**Affiliations:** 1 Creighton University, Omaha, NE, USA; 2 University of Iowa, Iowa City, IA, USA; 3 UNMC, Omaha, NE, USA

## Introduction

Trauma patients are subjected to higher radiation exposure (RE) as a function of Injury Severity Score (ISS) [1]. Analysis of this dataset was done to ascertain if RE varied with patient age.

## Methods

Data collection was as previously described [1]. RE was measured in milliSieverts (mSv). RE as a function of the ISS and age was explored for 7,661 trauma patients. The ISS groupings were as follows: ISS 1 to 8, ISS 9 to 15, ISS 16 to 25, and ISS >25. Age groupings were 19 to 39, 40 to 59, 60 to 79, and >80 years.

## Results

The data were analyzed in a hierarchical fashion: the mean exposure by age group was compared by one-sided, two-sample *t *tests. Differences were set up to test if mean exposure decreased with increasing age. Bonferroni adjustment was used for all multiple comparisons to maintain the overall error rate at 0.05. Of the six independent difference tests conducted, five were significant: 19 to 39 versus 60 to 79; 19 to 39 versus >79; 40 to 59 versus 60 to 79; 40 to 59 versus >79; and 60 to 79 versus >79 (*P *< 0.01). Analysis was done for ISS categories: ISS 1 to 8, ISS 9 to 15, ISS 16 to 25, and ISS >25. For ISS 1 to 8, five differences were significant: 19 to 39 versus 60 to 79; 19 to 39 versus >79; 40 to 59 versus 60 to 79; 40 to 59 versus >79; and 60 to 79 versus >79 (*P *< 0.01). For ISS 9 to 15, all differences were significant: 19 to 39 versus 40 to 59; 19 to 39 versus 60 to 79; 19 to 39 versus >79; 40 to 59 versus 60 to 79; 40 to 59 versus >79; and 60 to 79 versus >79 (*P *< 0.01). For ISS 16 to 25, four differences were significant: 19 to 39 versus 60 to 79; 19 to 39 versus >79; 40 to 59 versus 60 to 79; and 40 to 59 versus >79 (*P *< 0.01). For ISS >25, none of the differences were significant.

## Conclusion

Previous studies show significant RE in trauma patients [1]. We demonstrated the significance of RE to trauma patients, that the amount of RE has gone up chronologically over time, and that patients with an increasing ISS have a higher RE. We sought to determine whether age played a factor in RE. Based on our statistical analyses, older patients receive less RE for a given ISS. Although this is a large assessment of RE and trauma patients broken down by ISS and patient age, data analysis by year was limited by the small number of patients in high ISS groups at higher ages.
